# Detection and Characterization of Shiga Toxin Producing *Escherichia coli*, *Salmonella* spp., and *Yersinia* Strains from Human, Animal, and Food Samples in San Luis, Argentina

**DOI:** 10.1155/2014/284649

**Published:** 2014-08-07

**Authors:** Gabriela Isabel Favier, Cecilia Lucero Estrada, Teresa Inés Cortiñas, María Esther Escudero

**Affiliations:** General Microbiology, Faculty of Chemistry, Biochemistry and Pharmacy, National University of San Luis, Ejército de los Andes 950, Bloque 1 Piso 1, 5700 San Luis, Argentina

## Abstract

Shiga toxin producing *Escherichia coli* (STEC), *Salmonella* spp., and *Yersinia* species was investigated in humans, animals, and foods in San Luis, Argentina. A total of 453 samples were analyzed by culture and PCR. The antimicrobial susceptibility of all the strains was studied, the genomic relationships among isolates of the same species were determined by PFGE, and the potencial virulence of *Y. enterocolitica* strains was analyzed. *Yersinia* species showed higher prevalence (9/453, 2.0%, 95% CI, 0.7–3.3%) than STEC (4/453, 0.9%, 95% CI, 0–1.8%) and *Salmonella* spp. (3/453, 0.7%, 95% CI, 0–1.5%). *Y. enterocolitica* and *Y. intermedia* were isolated from chicken carcasses (6/80, 7.5%, 95% CI, 1.5–13.5%) and porcine skin and bones (3/10, 30%, 95% CI, 0–65%). One STEC strain was recovered from human feces (1/70, 1.4%, 95% CI, 0–4.2%) and STEC *stx1/stx2* genes were detected in bovine stools (3/129, 2.3%, 95% CI, 0–5.0%). *S*. Typhimurium was isolated from human feces (1/70, 1.4%, 95% CI, 0–4.2%) while one *S*. Newport and two *S*. Gaminara strains were recovered from one wild boar (1/3, 33%, 95% CI, 0–99%). The knowledge of prevalence and characteristics of these enteropathogens in our region would allow public health services to take adequate preventive measures.

## 1. Introduction

The detection and characterization of Shiga toxin producing* Escherichia coli* (STEC),* Salmonella* spp., and* Yersinia enterocolitica* strains in human patients, animal reservoirs, and foods of animal origin intended for human consumption are relevant to public health. These organisms are transmitted through contaminated drinking water and food and can cause intestinal and extraintestinal clinical manifestations in humans [1, 2]. STEC is associated with hemorrhagic colitis and hemolytic uremic syndrome (HUS); its pathogenicity is attributed to virulence factors that facilitate effective colonization of the human gastrointestinal tract and subsequent release of Shiga toxins [3]. Argentina has the highest incidence of HUS in the world, with* E. coli* O157:H7 as the primary etiological agent [4]. Previous studies in our country have been focused on the prevalence of STEC in cattle and byproducts intended for human consumption [4].

*Salmonella* spp. can enter the food chain, thereby triggering either sporadic cases or outbreaks of human salmonellosis [5]. Foodborne outbreaks triggered by Salmonella serotypes are of rare occurrence in our province; however,* S*. Panama,* S*. Newport,* S*. Sandiego,* S*. Enteritidis,* S*. Montevideo,* S*. Anatum, and* S*. Typhimurium strains have been isolated from sources of diverse origin [6].

Pigs are the most important* Y. enterocolitica* carriers. Pathogenic strains usually carry a 72 kb virulence plasmid (pYV) bearing y*adA*,* yops*, and* virF* genes and chromosomal virulence markers such as* ail*,* yst*,* myfA*, and* inv* genes [7]. The assessment of virulence markers contributes to properly establish the pathogenic potential of the* Y. enterocolitica* isolates. Since* Y. enterocolitica* is not routinely investigated in the clinical laboratories of Argentina, a systematic epidemiological study of* Y. enterocolitica* in environment, reservoirs, foods, and humans is still pending.

For contributing to the knowledge of prevalence and distribution of these enteropathogens in patients, presumable animal reservoirs, and foods of our region, this study was aimed (i) to detect STEC,* Salmonella* spp., and* Y. enterocolitica* in human and animal feces and foods of animal origin intended for human consumption, (ii) to assess the pathogenic potential of* Y. enterocolitica* strains through phenotypic and molecular virulence markers, (iii) to test the antimicrobial susceptibility of all strains, and (iv) to determine possible genetic relationships among isolates of each species by subtyping using pulsed field gel electrophoresis (PFGE). In addition, counts of total coliforms were performed in samples of foods.

## 2. Materials and Methods

### 2.1. Bacterial Strains

*Y. enterocolitica* O:9 W1024 pYV^+^ (Belgium),* Y. enterocolitica* MCH700 B4 O:3 pYV^+^ (Canada),* Y. enterocolitica* 29C-43 B4 O:3 pYV^+^ (Norway), and* Y. enterocolitica* B2 O:9 pYV^+^ (Argentina) were used as positive controls in phenotypic virulence assays, assessment of genetic virulence markers by PCR and comparison of DNA profiles by PFGE.* Escherichia coli* O157:H7 EDL933* Sor-/*β*glu-/E-Hly+/eae*+, biotype C, producer of Stx1 and Stx2, was employed as positive control in PCR targeting STEC* stx1/stx2* genes and* Salmonella* Braenderup H9812 was used as molecular size marker in PFGE.* Y. enterocolitica* B1A O:5 CLO229 and* Y. enterocolitica* B1A O:6,30 CLO225, local strains isolated from sausages, were utilized as positive controls in* ystB* gene PCR [8]. These strains were maintained at 4°C on trypticase soy agar slants (TSA, Merck, Darmstadt, Germany). For determining culture purity, isolations on Mac Conkey agar (MC, Merck), Sorbitol Mac Conkey agar (SMAC, Merck), and Salmonella Shigella agar (SS, Merck) for* Y. enterocolitica, E. coli* O157:H7, and* S. *Braenderup, respectively, were made prior to each experiment. Plates were incubated 48 h at 25°C for* Y. enterocolitica* and 24 h at 37°C for* E. coli* O157:H7 and* S*. Braenderup.

### 2.2. Samples

A total of 453 samples from human and animal sources in San Luis city and upcoming rural areas, Argentina, were analyzed during the period June 2008–November 2011. They included 70 samples of human stools from patients with enterocolitis symptoms attending a local clinical laboratory; 167 stool samples obtained from feedlot bovines (*n* = 61), grazing bovines (*n* = 68), porcine (*n* = 20), ovine (*n* = 10), goats (*n* = 6), and equines (*n* = 2); and 216 samples of animal origin intended for human consumption such as chicken carcasses (*n* = 80), porcine skin and bones (*n* = 10), goat cheeses (*n* = 30), fresh sausages “chorizos” (*n* = 90), and six samples of three wild boars, tongues (*n* = 3), and tonsils (*n* = 3). Samples of animal stools were randomly collected in regional cattle markets and farms immediately after defecation. Samples of wild boars were hunt products from our region. Samples of foods of animal origin intended for human consumption were purchased at five retail markets in San Luis city. All samples were packed in individual sterile plastic bags and stored at 4°C for up to 6 h before processing.

### 2.3. Investigation of Total Coliforms

The presence of total coliforms was investigated in samples of animal origin intended for human consumption, except tonsils and tongues of wild boars. Decimal dilutions of each sample were prepared in 0.1% peptone water pH 7.2 (PW, Merck); one milliliter of each dilution was seeded in violet-lactose-neutral red-bile agar (VLRB, Merck) and incubated at 37°C for 24 h. Counts of characteristic colonies were performed and results were expressed as log_10_ CFU/g.

### 2.4. Investigation of Shiga Toxin-Producing* Escherichia coli* (STEC),* Salmonella* spp., and* Y. enterocolitica*

#### 2.4.1. STEC

Samples of 25 g were homogenized in stomacher (IUL Masticator, Germany) for 90 s and incubated in 225 mL of EC broth (Merck) for 24 h at 37°C. For avoiding false negative PCR results produced by interference of organic compounds and background microflora present in samples,* stx1/stx2* PCR was not immediately performed. Instead, EC enriched samples were streaked on SMAC incubating 24 h at 37°C [9]. Each plate showed confluent growth in the initial streaking zone where bacteria were clustered and individual colonies in the final streaking zone where bacteria were isolated. Then, DNA was extracted from confluent growth for screening* stx1/stx2* genes by PCR [9]; if amplified, five sorbitol nonfermenting and five fermenting colonies were randomly selected from SMAC plates and their DNA were individually tested by PCR targeted to* stx1* and* stx2* genes. When positive results were observed, Gram staining and biotyping of each colony was performed. Sorbitol nonfermenting* E. coli* colonies were challenged against O157 antiserum (National Institute of Infectious Diseases-INEI ANLIS-“Dr. Carlos G. Malbrán,” Buenos Aires, Argentina) by a slide agglutination test.

#### 2.4.2. *Salmonella* ****spp.****

This microorganism was investigated according to U.S. FDA Bacteriological Analytical Manual (http://www.fda.gov/Food/FoodScienceResearch/LaboratoryMethods/ucm2006949.htm, accessed in May 2008). Twenty five grams of each homogenized sample was enriched in 225 mL of lactose broth (LB, Merck) at 37°C for 24 h; then, one-mL LB aliquots was transferred into two tubes containing 9 mL of tetrathionate broth (Merck) and two tubes with 9 mL of Rappaport-Vassiliadis broth (Merck). One tube of each selective broth was incubated at 37°C for 24 h and the other one was incubated at 42°C for 24 h. Isolations were done on SS agar (Merck) for 24 h at 37°C and suspect lactose nonfermenting colonies were examined by Gram staining and biochemical assays. Serotyping was performed in the National Reference Center for Enterobacteria, INEI-ANLIS, Buenos Aires, Argentina.

#### 2.4.3. *Y. enterocolitica*

This species was searched by enrichment of 25-g homogenized sample in 225 mL of phosphate buffered saline pH 7.6 added with 1% sorbitol and 0.15% bile salts and incubated 21 days at 4°C. Isolations were made on MC agar for 48 h at 25°C and presumptive* Yersinia* colonies were examined by Gram staining and identified by biochemical assays (http://www.fda.gov/Food/FoodScienceResearch/LaboratoryMethods/ucm072633.htm, accessed May 2008). The final characterization in biotypes and serotypes was performed by Dr. E. Carniel, National Reference Center of* Yersinia*, Institute Pasteur, Paris, France.

### 2.5. PCR Targeting STEC* stx1* and* stx2* Genes

DNA extraction was performed by the boiling method [9]. Each strain was suspended in an Eppendorf tube containing 150 *μ*L of TE 1X buffer (10 mM Tris (Sigma Aldrich, St. Louis, MO, USA), 1 mM EDTA (Sigma), pH 8.0) added with 1% Triton-X100 (Parafarm, Buenos Aires, Argentina). Suspensions were boiled 15 min and centrifuged at 10,000 rpm for 5 min (Sigma centrifuge model 3K30, Germany). Each supernatant containing DNA was transferred to other tube and stored at −20°C before use. A 25 *μ*L volume of a PCR mix containing 1X PCR buffer, 0.1 mM dNTP, 1.5 mM MgCl_2_, 2 pmol/*μ*L of* stx1* primers (forward 5′-GAAGAGTCCGTGGGATTACG-3′ and reverse 5′-AGCGATGCAGCTATTAATAA-3′) for an amplicon of 130 bp, 0.4 pmol/*μ*L of* stx2* primers (forward 5′-TTAACCACACCCCACCGGGCAGT-3′ and reverse 5′-GCTCTGGATGCATCTCTGGT-3′) for an amplicon of 346 bp, 0.02 U/*μ*L Taq DNA polymerase (Productos Biológicos, Quilmes, Argentina), 2 *μ*L DNA template, and ultrapure water was prepared. Cycling conditions consisted of an initial denaturation at 94°C for 5 min followed by 30 cycles of 94°C for 30 s, 58°C for 30 s, and 72°C for 30 s and a final extension of 72°C for 3 min [9]. PCR was performed in a Techne TC-512 thermal cycler (Techne Inc., Duxford, UK). The PCR products were electrophoresed in a 2% agarose gel (Productos Biológicos) added with GelRed Acid Gel Stain (Biotium, Hayward, CA, USA), 1.5 *μ*L stock solution/40 *μ*L gel, at 80 V for 40 min, and visualized in an UV transilluminator (UVP, Upland, CA, USA). For comparison, a 100-bp DNA ladder (Productos Biológicos) was used.

### 2.6. PFGE

DNA preparation was according to Ribot et al. [10]. Each bacterial strain was isolated on Mueller Hinton agar (MH, Britania, Buenos Aires, Argentina) and incubated 24 h at 37°C for* Salmonella* and* E. coli*, or 48 h at 25°C for* Yersinia* strains. Colonies of each strain were directly suspended in 4 mL of the suspension buffer (100 mM Tris, 100 mM EDTA, pH 8.0) to OD_610 nm_ 1.0. Two hundred microliters of bacterial suspension were mixed with equal volume of 1% SeaKem Gold agarose (Cambrex, Rockland, ME, USA) and poured in molds to obtain “plugs.” Plugs were treated for 20 h in a lysis solution (50 mmol Tris-EDTA (Sigma), 1% sodium lauroyl sarcosine (Sigma), and Proteinase K 0.1 mg/mL (Fluka Chemie, Buchs, Switzerland) pH 8.0) at 37°C and then washed four times with TE buffer (10 mM Tris (Sigma), 1 mM EDTA (Sigma), pH 8.0) for 30 min at 37°C. Chromosomal DNA contained in agarose plugs was digested with 10 U* XbaI* (Fermentas, Burlington, Ontario, Canada) for two hours, according to manufacturer's instructions. Plugs were cut in approximately 1-mm-thick slices, placed in the CHEF-DRIII chamber (Bio-Rad, Hercules, CA, USA), and PFGE was performed using an electric field of 6 V/cm at 14°C, angle of 120° and switching times of 2.2 (initial time) to 63.8 s (final time) for* Salmonella* and* E. coli* O157:H7, and 1.8 s (initial time) to 20 s (final time) for* Y. enterocolitica*, over 20 h [6, 8]. Migration of the DNA fragments was achieved in a 1.0% pulsed-field agarose gel (Bio-Rad) submerged in 0.5X TBE buffer (45 mM Tris-borate (Sigma) and 1 mM EDTA (Sigma)). The gels were stained with Gel Red Acid Gel Stain (Biotium, Hayward, CA, USA) under the conditions suggested by the manufacturer, visualized in UV transilluminator, and photographed. The size standard strain was* S.* Braenderup H9812, kindly donated by Dr. N. Binsztein (INEI-ANLIS, Buenos Aires, Argentina).

### 2.7. Virulence Phenotypical Assays of* Y. enterocolitica* Isolates

These assays were performed according to US FDA Bacteriological Analytical Manual (http://www.fda.gov/Food/FoodScienceResearch/LaboratoryMethods/ucm072633.htm, accessed September 2011).

#### 2.7.1. Autoagglutination

Each strain was inoculated into two tubes of Methyl Red-Voges Proskauer broth (Merck). One tube was incubated at 37°C and the other was incubated at 25°C. After 18 to 24 h, the tubes were observed for bacterial autoagglutination The MR-VP tube incubated at 25°C for 24 h should show turbidity from bacterial growth. The 37°C MR-VP tube should show agglutination (clumping) of bacteria along walls and/or bottom of tube with clear supernatant fluid. Isolates giving this result were presumptive positive for the virulence plasmid. Any other pattern for autoagglutination at these two temperatures was considered negative.

#### 2.7.2. Calcium Dependence at 37°C and Congo Red Binding

Congo Red-Magnesium Oxalate sodium agar (CR-MOX) was composed by TSA added with 2.68 g/L sodium oxalate (Mallinckrodt), 4.067 g/L MgCl_2_·H_2_O (Sigma), and 5 mL/L of 1% Congo Red dye (Baker), which allowed visualization of calcium-dependent growth and uptake of Congo Red dye on the same plate. The test organism was inoculated into TSB and incubated overnight at 25°C. Decimal dilutions in physiologic saline were made to obtain 1,000 cells/mL. Volumes of 0.1 mL of the appropriate dilution were spread-plated on CR-MOX plates and incubated at 37°C. Presumptive plasmid-bearing* Y. enterocolitica* showed scarce growth of pinpoint, round, convex, red, and opaque colonies; whereas plasmidless* Y. enterocolitica* exhibited abundant growth of large, irregular, flat, and translucent colonies.

#### 2.7.3. Esculin Hydrolysis

This test was performed on esculin agar (10 g polypeptone (Britania), 1 g esculin (Sigma), 1 g ferric ammonium citrate (Sigma), and 5 g agar (Britania) in 1000 mL of distilled water). Strains were inoculated, incubated at 25°C, and observed up to 7 days after seeding. The development of dark pigment on the agar surface was indicative of lacking of virulence.

#### 2.7.4. Pyrazinamidase Production

Strains were inoculated over the entire slant of pyrazinamide agar (30 g TSA (Merck), 1 g pyrazinecarboxamide (Sigma), and 0.2 M Tris-maleate buffer (Sigma) for 1000 mL), incubated at 25°C for 48 h, and flooded with 1 mL of 1% freshly prepared ferrous ammonium sulfate. Development of pink color within 15 min was positive test, indicating presence of pyrazinoic acid formed by the pyrazinamidase enzyme.

### 2.8. PCR for* Y. enterocolitica* Molecular Virulence Markers (*yadA* and* ystB* Genes)

#### 2.8.1. Nested* yadA* PCR

DNA extraction was performed as described [9]. A nested-PCR targeted to* Y. enterocolitica yadA* gene [11] was applied using two sets of primers. Five microliters of the template was used for the first PCR and 2 *μ*L of the product obtained in this step was used as template for the second PCR. The reaction mixture (50 mL) contained 1X PCR buffer, 1.5 mM MgCl_2_, 200 mmol/mL of each dNTP, 1 U of Taq DNA-polymerase, and 0.1 mmol/mL of each primer (Productos Biológicos). The first primer pair integrated by the following oligonucleotides: YadA1 5′-TAA GAT CAG TGT CTC TGC GGC A-3′ and YadA2 5′-TAG TTA TTT GCG ATC CCT AGC AC-3′ was used under the following cycling conditions: initial denaturation at 95°C for 3 min, then 40 cycles of denaturation at 95°C for 30 s, annealing at 58°C for 60 s, and extension at 72°C for 90 s, followed by a final extension at 72°C for 10 min for amplifying a 747 bp fragment. The second primer pair consisting of YadA3 5′-GCG TTG TTC TCA TCT CCA TAT GC-3′ and YadA4 5′-GGC TTT CAT GAC CAA TGG ATA CAC-3′ was used under the following cycling conditions: initial denaturation at 95°C for 3 min followed by 20 cycles of denaturation at 95°C for 30 s, annealing at 62°C for 60 s, and extension at 72°C for 90 s, and a final extension at 72°C for 10 min which amplified a 529 pb fragment. Products were electrophoresed at 80 V for 40 min in a 1% agarose gel stained with GelRed, visualized in an UV transilluminator (UVP), and compared with a 100-bp ladder DNA (Productos Biológicos).

#### 2.8.2. Simple* ystB* PCR

DNA extraction was performed by the boiling method [9]. The reaction mixture (25 *μ*L) contained PCR buffer 1X, 200 *μ*M of each dNTP, 1.5 mM MgCl_2_, 0.08 U/*μ*L of Taq DNA-polymerase, and 1 pmol/*μ*L of each primer (Productos Biológicos). It used the primer pair constituted by YstB-F 5′GTACATTAGGCCAAGAGACG 3′ and YstB-R 5′ GCAACATACCTCACAACACC 3′ for amplifying a 146 bp fragment [12]. The PCR product was electrophoresed at 80 V for 40 min in a 2% agarose gel stained with GelRed and visualized in an UV transiluminator (UVP). The molecular mass of amplicons was determined as described above.

### 2.9. Antimicrobial Susceptibility

The antimicrobial susceptibility of the isolates was addressed by the disk diffusion method on Mueller Hinton agar. The following antibiotic disks (Britania) were used: ampicillin, 10 *μ*g; cephalotin, 30 *μ*g; aztreonam, 30 *μ*g; erythromycin, 15 *μ*g; colistin 10 *μ*g; chloramphenicol, 30 *μ*g; gentamicin, 10 *μ*g; tetracycline, 30 *μ*g; trimethoprim-sulfamethoxazole, 25 *μ*g; ciprofloxacin, 5 *μ*g; neomycin, 30 *μ*g; furazolidone, 5 *μ*g; nalidixic acid, 30 *μ*g; cefotaxime, 30 *μ*g; ceftriaxone, 30 *μ*g; and phosphomycin, 50 *μ*g. Zones of growth inhibition were evaluated according to Clinical and Laboratory Standards Institute (CLSI) guidelines [13].

### 2.10. Statistical Analysis

Statistical analysis of counts of coliforms* related to type of sample* was performed using Chi-square test (Analytical Software, Tallahassee FL, USA). Statistical calculations were based on confidence level equal or higher than 95% (*P* ≤ 0.05 was considered statistically significant). Confidence intervals for each percentage were calculated by using the formula: 95% CI = percentage ± (1.96 × SE), with SE, standard error, calculated as SE = percentage/(N° of positive cases)^1/2^. The discrimination index (DI) values of PFGE were calculated by Simpson's diversity index. Clustering of the patterns obtained by PFGE was performed using Statistica 6.0 software (StatSoft Inc., Tulsa, OK, USA) and the unweighted pair group method with arithmetic average (UPGMA).

## 3. Results

### 3.1. Investigation of Total Coliforms, Shiga Toxin-Producing* Escherichia coli* (STEC),* Salmonella* spp., and* Y. enterocolitica*

Counts of total coliforms in fresh sausages and chicken carcasses yielded 5.4 ± 0.7 and 5.0 ± 0.7 log_10_ CFU/g, respectively, being significantly higher (*P* ≤ 0.05) than those observed for porcine skin and bones (3.9 ± 0.6 log_10_ CFU/g) and goat cheeses (0.7 ± 0.2 log_10_ CFU/g) (Table 1). The detection frequencies of STEC,* Salmonella* serotypes, and* Yersinia* species in 453 samples are shown in Table 2. The number of* Yersinia* positive samples (9/453, 2.0%, 95% CI, 0.7–3.3%) was higher (*P* ≤ 0.05) than those observed for STEC (4/453, 0.9%, 95% CI, 0–1.8%) and* Salmonella* spp. (3/453, 0.7%, 95% CI, 0–1.5%).

A sample was considered “STEC positive” when* E. coli* colonies carrying* stx1/stx2* genes could be recovered by culturing, or “presumptive STEC positive” when only* stx1/stx2* signals were detected by PCR on DNA extracted from confluent growth on SMAC. Thus, four samples were positive: one stool sample from a pediatric patient with diarrhea (1/70, 1.4%, 95% CI, 0–4.2%) yielded one* E. coli* O157:H7 strain by culture on SMAC which was characterized as* stx2* ^*+*^ by PCR and three samples of bovine stools (3/129, 2.3%, 95% CI, 0–5.0%) that amplified* stx* genes from DNA extracted from confluent growth (two samples were* stx1* ^*+*^/*stx2* ^*+*^and the third one was* stx2* ^+^). Individual STEC colonies could not be isolated from these samples.

On the other hand, three samples yielded four* Salmonella* isolates corresponding to different serotypes: one* S*. Typhimurium strain was obtained from stools of a symptomatic patient (1/70, 1.4%, 95% CI, 0–4.2%) and one* S*. Newport and two* S*. Gaminara strains were isolated from one tonsil and one tongue of wild boar among 216 samples of animal origin intended for human consumption (2/216, 0.9%, 95% CI, 0–2.1%). In the small number of wild boar samples, the* Salmonella* recovery was high (1/3, 33%, 95% CI, 0–99%). Interestingly, the tonsil positive sample carried both* S*. Gaminara and* S*. Newport strains. Furthermore, nine* Yersinia* isolates were recovered from nine (9/216, 4.2%, 95% CI, 1.5–6.9) samples of animal origin intended for human consumption. Two isolates were classified as* Y*.* enterocolitica* B1A O:12,25-12,26, five as* Y. enterocolitica* B1A O:7,8-8-8,19, one as* Y. intermedia* B6 O:17, and other one as* Y. intermedia* B4 O:40 (Table 2). They were isolated from six chicken carcasses (6/80, 7.5%, 95% CI, 1.5–13.5%) and three porcine skin and bones (3/10, 30%, 95% CI, 0–65%).

### 3.2. PFGE

**Figure 1:**
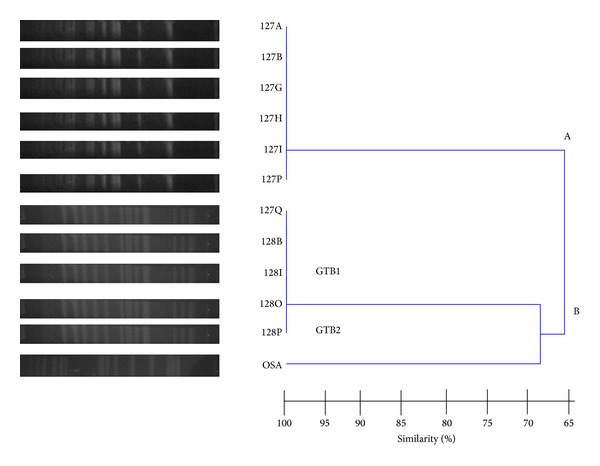
Fingerprints and dendrogram obtained by PFGE of twelve* Salmonella* isolates in this study. GT, genomic type. Six* S. *Newport (127A, B, G, H, I, and P), and one* S.* Gaminara (127Q) isolates from a tonsil, and four* S. *Gaminara (128B, I, O, and P) isolates from tongue of the same wild boar.* S. *Typhimurium strain of human origin (OSA).

*XbaI*-restricted DNA polymorphisms of* Salmonella* isolates are observed in Figure 1. Two major clusters, A and B, with a 65% similarity were obtained. Even though six* S*. Newport isolates from tonsil and five* S*. Gaminara isolates from tonsil and tongue were initially recovered from one wild boar, the analysis of their DNA restriction profiles by PFGE showed that all* S*. Newport strains grouped in cluster A while all* S*. Gaminara ones grouped in the genotype GTB1 within cluster B. Since identical DNA band patterns between isolates of the same serovar were observed, only three* Salmonella* strains were reported (Table 2). In dendrogram,* S*. Typhimurium of human source was included in GTB2 within cluster B, showing 68% similarity with GTB1.

**Figure 2:**
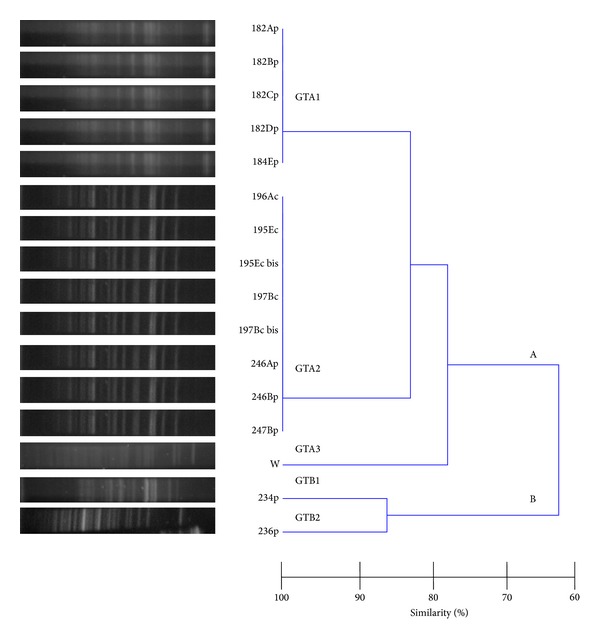
Fingerprints and dendrogram obtained by PFGE of fifteen* Yersinia* isolates. GT, genomic type.* Y. enterocolitica* B1A O:12,25-12,26 (182Ap, 182Bp, 182Cp, 182Dp, 184Ep) isolates from two chicken carcasses;* Y. enterocolitica* B1A O:7,8-8-8,19 isolates from three (196Ac, 195Ec, 195Ec bis, 197Bc, 197Bc bis) porcine skin/bones and two (246Ap, 246Bp, 247Bp) chicken carcasses;* Y. enterocolitica* W1024 reference strain (W);* Y. intermedia* B6 O:17 (234p) and* Y. intermedia* B4 O:40 (236p).

Although fifteen* Yersinia* isolates were originally recovered from nine positive samples, the analysis of DNA restriction profiles observed by PFGE allowed to conclude that some isolates were replicates of the same strain. Therefore, bacterial isolates were grouped into two major clusters, A and B (63% similarity), according to* Yersinia* species (Figure 2). Thus, cluster A comprised GTA1 with five* Y. enterocolitica* B1A O:12,25-12,26 strains isolated from chicken carcasses, GTA2 with eight* Y. enterocolitica* B1A O:7,8-8-8,19 isolates from chicken carcasses and porcine skin, and GTA3 with the reference* Y. enterocolitica* W1024 strain. Cluster B included GTB1 consisting of* Y. intermedia* B6 O:17 strain and GTB2 corresponding to* Y. intermedia* B4 O:40 strain (86% similarity), both strains recovered from chicken carcasses. PFGE was not applied on the human STEC strain isolated in this study.

### 3.3. Phenotypic and Molecular Virulence Assays of* Y. enterocolitica* Isolates

Five* Y. enterocolitica* B1A O:7,8-8-8,19 strains and two* Y. enterocolitica* B1A O:12,25-12,26 strains isolated in this study produced negative results for calcium dependent growth and Congo red binding at 37°C showed pyrazinamidase activity, hydrolyzed esculin and autoagglutinated at 37°C (Table 3). Nested PCR targeting* yadA* gene yielded negative results in all cases. When* ystB* gene was assayed, one* Y. enterocolitica* B1A O:7,8-8-8,10 strain isolated from porcine skin and bones was positive.

### 3.4. Antimicrobial Susceptibility

The human STEC strain was susceptible to all antimicrobials assayed except ampicillin and rifampicin. Similarly,* Salmonella* strains were susceptible to all drugs except cephalotin. Meanwhile, all* Y. enterocolitica* isolates shared resistance to ampicillin, one* Y. enterocolitica* B1A O:12,25-12,26 strain was susceptible to cephalotin, and two* Y. enterocolitica* B1A O:7,8-8-8,10 strains showed resistance to both cephalotin and erythromycin.

## 4. Discussion

Total coliforms are considered indicators of hygienic quality and their presence in foods may correlate with the presence of pathogenic bacteria. The low total coliform counts observed in porcine skin and bones, and goat cheeses might be attributed to the effects of thermal treatments applied to pig carcasses during slaughtering, and pasteurization and preservation of dairy products, respectively. No microbiological specifications for porcine skin and bones are included in the Argentinean Alimentary Code (AAC, http://www.anmat.gov.ar/alimentos/normativas_alimentos_caa.asp, accessed November 2013). On the other hand, values up to 500 total coliforms per gram at 45°C are allowed for cheeses with 36 to 46% moisture (AAC). Thus, low coliform counts for this food would be consistent with good practices of manufacture. In contrast, low microbiological quality of ingredients or poor hygiene could explain coliform counts higher than 10^3^ MPN/g which is the maximal limit established by AAC for fresh sausages. Although no microbiological standards for chicken carcasses are addressed by AAC, counts of coliforms in this work were higher than 2.7 log_10_ CFU/g observed by Capita et al. [14] in Spain. Clearly, contamination is possible at any stage of the production process, from defeathering, evisceration, and washing to storage by cooling or freezing.

Regarding the search of enteropathogens, the human* E. coli* O157:H7 strain was isolated by culture and characterized as* stx2* ^*+*^ by PCR. On the contrary, no STEC strain could be isolated from positive* stx1/stx2* cattle stools, probably because they were viable but noncultivable strains. Concurrently, Jure et al. [15] identified the* stx2* gene in seven samples of meat in Argentina; however, only one* E. coli* O157:H7 strain could be isolated. The low detection of STEC from cattle in San Luis contrasts with reports of 4 to 39% STEC isolates recovered from calves by Meichtri et al. [16] in our country, who enriched stools and rectal swabs in TSB added with antibiotics and then performed screening of* stx* genes by conventional PCR in DNA extracted from confluent bacterial growth on SMAC. If amplified, PCR was repeated on individual colonies. Similarly, Sanz et al. [17] recovered 44% STEC from bovines for slaughtering in other Argentina regions. A wide range of protocols have been described for detection or isolation of STEC since that all serotypes cannot be detected by one method [18]. Trypticase soy broth,* E. coli* broth, buffered peptone water, and brain heart infusion broth added with selective agents have been recommended for STEC enrichment. In the present study, samples were enriched in EC broth without antibiotics which may be advisable when stressed or injured STEC cells are cultured [19]. In addition, a comparative study of enrichment protocols by Vimont et al. [20] showed that the initial level of* E. coli* O157 was not greatly influenced by the enrichment protocol tested, whereas the initial level of background microflora appeared to decrease when EC broth was used. Other techniques have been recommended for improving the sensitivity of detection methods. The immunomagnetic separation (IMS) can be used after enrichment and prior to plating for the selective concentration of STEC cells, and it is well established for the detection of* E. coli* O157 in foods, yielding detection limits as low as 1-2 CFU/25 g [18]. While IMS was not used in this study, subsequent STEC researches in our laboratory will include this procedure. Otherwise, molecular methods such as conventional PCR and real-time PCR are very sensitive and provide results in shorter times than cultures. Thus, the ISO/TS 13136:2012 standard is based on the sample enrichment followed by a real-time PCR targeted to the detection of the* stx* and* eae* virulence genes, and the determination of O157, O111, O26, O103, and O145 STEC serogroups in foods and animal foodstuffs. When genes are detected, the STEC strain should be isolated for confirmation [18]. Also, immunoassay-based methods such as an available EIA for testing Shiga toxins 1 and 2 have been used in the STEC detection from human stools [21]. Differences in STEC carriage have been observed between grass-fed and feedlot cattle [22]; in the present study, two positive* stx1/stx2* samples corresponded to feedlot animal and the other one came from a grazing animal. Although STEC detection and/or recovery were negative in other samples studied here, Ojo et al. [23] demonstrated STEC in feces of cattle (15.2%), sheep (10.7%), goats (7.5%), and pigs (5.6%) as well as in beef (3.8%), goat-meat (1.7%), and pork (4.0%).

The isolation of* S. *Typhimurium from stools of a patient was consistent with studies reporting this one as the most frequently isolated serovar from humans in Argentina since 2006 [24]. We report the isolation of* S.* Newport and* S. *Gaminara from wild boars for the first time in our region. These* Salmonella* serotypes have been previously isolated from clinical samples during an outbreak caused by consumption of unpasteurized orange juice in USA [25] and recovered from patients with diarrhea in Caribbean zone of Colombia [26]. Environmental factors and seasonal variations as well as different supply sources of samples might have influenced in the low recovery of* Salmonella* from animal samples in our study.

The* Yersinia* prevalence observed in this work was lower than 5.5% from pork and beef sausages and minced meat obtained by Lucero Estrada et al. [8] who detected* Y. enterocolitica* B1A (O:5 and O:6,30), B2 O:9, and* Y. intermedia* in our region. Previously, Floccari et al. [27] isolated 10%* Y. enterocolitica* B1A O:5,* Y. intermedia*, and* Y. frederiksenii* from 70 chicken carcasses in Argentina. AAC establishes no* Y. enterocolitica* limits in relation to any of the foods here analyzed, but the absence of this pathogen is desirable. Although* Y. enterocolitica* was not detected in human and animal stools investigated in the present study, this microorganism has been isolated from human diarrheic feces [28] and animal stools [29] in our country.

In this study, virulence phenotypic tests for* Y. enterocolitica* B1A strains produced negative results excepting autoagglutination at 37°C; however, the* yadA* gene was not detected by PCR. Lack of correlation between* Y. enterocolitica* phenotypic and genotypic virulence markers such as the above mentioned has been reported by Zheng et al. [30]. These authors found that some* Y. enterocolitica* strains contain other unknown virulence markers that interact with each other and play an important role in the pathogenesis. In this regard, the chromosomal gene* ystB* had been strongly linked to the production of diarrhea by B1A strains. Opportunely, among 115* Y. enterocolitica* isolates of pig origin analyzed by Bonardi et al. [31], 75.7% corresponded to B1A with* ystB* as the most common virulence gene (72.4%). In our study, this gene was demonstrated in one* Y. enterocolitica* B1A O:7,8-8-8,10 strain (1/7, 14%, 95% CI, 0–42%) isolated from porcine skin and bones. The presence of* Y. enterocolitica* B1A and* Y. intermedia* in chicken carcasses and porcine skin and bones could be the result of cross-contamination during processing of these products or carriage by slaughtered pigs, respectively.

Related to STEC antimicrobial susceptibility, since antimicrobials can injure the bacterial membrane causing an acute release of preformed Shiga toxin [32], the treatment of HUS in patients is mostly supportive with adequate corporal fluid and electrolyte management, control of the haematological complications, antihypertensive and analgesic therapy, mechanical ventilation, and dialysis when necessary [33], avoiding antibiotic administration. In our region, STEC strains isolated from patients with diarrhea have demonstrated* in vitro* susceptibility to antibiotics commonly used in the treatment of infections triggered by other enterobacteria. Contrary to the antibiotic sensitivity demonstrated by our* Salmonella* isolates, Ibar et al. [34] observed multidrug resistance in different* Salmonella* serotypes isolated from porcine in Argentina against antimicrobials commonly used in veterinary medicine. Regarding* Y. enterocolitica* antimicrobial susceptibility, our results matched those reported by Lucero Estrada et al. [8] and Bonardi et al. [31] who observed resistance to cephalotin and ampicillin.

## 5. Conclusions

A low prevalence of STEC,* Salmonella* spp., and* Yersinia* species was observed in human, animal, and food samples in this region of Argentina. The low number of STEC found in this study, one* E. coli* O157:H7 from human stool, as compared to other works, might be attributed to the detection methods used. Otherwise, the detection of* stx1/stx2* genes in cattle stools highlights the risk of exposure to STEC animal carriers and reinforces the requirement of the good practices of hygiene during slaughtering and meat processing. On the other hand, the high* Salmonella* frequency observed in the small number of wild boar samples emphasizes the need of further studies in these animals whose byproducts are manufactured and marketed at retail. Lastly, bio/serotypes and virulence traits characterizing our* Y. enterocolitica* isolates were related to null or low pathogenicity for humans; however, a wide field of knowledge remains unexplored about* Y. enterocolitica* B1A virulence. Our results suggest that a close microbiological monitoring might contribute to the knowledge of prevalence and distribution of these enteropathogens in patients, presumable animal reservoirs, and foods in our region, which would allow public health services to take preventive measures.

## Figures and Tables

**Table 1:** Counts of total coliforms in samples of animal origin purchased at retail markets and intended for human consumption.

Type of samples	Number of samples	log_10_⁡ CFU/g
		± SD^∗^
Chicken carcasses	80	5.0 ± 0.7^A^
Porcine skin and bones	10	3.9 ± 0.6^B^
Goat cheeses	30	0.7 ± 0.2^C^
Fresh sausages “chorizos”	90	5.4 ± 0.7^A^

Total	210	

^∗^SD: standard deviation.

^A,B,C^Values of log_10_⁡ CFU/g followed by different capital letters are statistically different (*P* ≤ 0.05). Counts were performed on violet red bile (VRB) agar.

**Table 2:** Frequency of detection of STEC, *Salmonella* serotypes, and *Yersinia* species in samples of diverse origin analyzed in this study.

Source	Number of samples	STEC		*Salmonella *spp.		*Yersinia* species		
		Positive samples (% ± 1.96SE)∗		Positive samples (% ± 1.96SE)∗	Serovar (number of strains)	Positive samples (% ± 1.96SE)∗	Species	Bioserovar (No. strains)
		Culture	PCR					
Human stools	70	1 (1.4 ± 2.8)	—	1 (1.4 ± 2.8)	*S. *Typhimurium (1)	—	—	
Animal stools:	167							
feedlot bovines	61	—	2 (3.3 ± 4.6)	—		—		
grazing bovines	68	—	1 (1.5 ± 3.0)	—		—		
porcines	20	—	—	—		—		
ovines	10	—	—	—		—		
goats	6	—	—	—		—		
equines	2	—	—	—		—		
Samples of animal origin for human consumption:	216							
chicken carcasses	80	—	—	—		6 (7.5 ± 6.0)	*Y. enterocolitica* *Y. intermedia *	B1A O:12,25-12,26 (2) B1A O:7,8-8-8,19 (2) B6 O:17 (1) B4 O:40 (1)
porcine skin and bones	10	—	—	—		3 (30 ± 35)	*Y. enterocolitica *	B1A O:7,8-8-8,19 (3)
goat cheeses	30	—	—	—		—		
fresh sausages	90	—	—	—		—		
wild boars	3							
tonsils	3	—	—	1 (33 ± 66)	*S. *Newport (1) *S.* Gaminara (1)	—		
tongues	3	—	—	1 (33 ± 66)	*S.* Gaminara (1)	—		

Total	453	4 (0.9 ± 0.9)^†^		3 (0.7 ± 0.8)^†^		9 (2.0 ± 1.3)^†^		

^∗^(%): percentage corresponding to positive samples/total samples of the same type; ^†^(%): percentage corresponding to total positive samples for each pathogen/total of samples. In both cases, 1.96SE is the *t* value (*α* 0.05) multiplied by the standard error.

**Table 3:** Phenotypic virulence assays corresponding to *Y. enterocolitica* strains.

Total of strains	Bioserotype	Origin	Phenotypic assays∗				
			Esc	Pyr	AA	Ca^2+^	CR
2	B1A O:12,25-12,26	Chicken carcasses	+	+	+	−	−
3	B1A O:7,8-8-8,19	Porcine skin and bones	+	+	+	−	−
2	B1A O:7,8-8-8,19	Chicken carcasses	+	+	+	−	−
1	W1024 B2 O:9	Reference strain	−	−	+	+	+
1	MCH 700 B4 O:3	Reference strain	−	−	+	+	+
1	29C-46 B4 O:3	Reference strain	−	−	+	+	+
1	B2 O:9	Eggshell (local strain)	−	−	+	+	+

^∗^Esc: esculin hydrolysis; Pyr: pyrazinamide hydrolysis; AA: autoagglutination at 37°C; Ca^2+^: calcium dependence; and CR: congo red binding.
